# Advancements in the diagnosis and treatment of renal epithelioid angiomyolipoma: A narrative review

**DOI:** 10.1002/kjm2.12586

**Published:** 2022-09-03

**Authors:** Jian‐Wei Yang, Cheng Liang, Li Yang

**Affiliations:** ^1^ Department of Urology The Second Hospital of Lanzhou University Lanzhou China; ^2^ Clinical Center of Gansu Province for Nephron‐urology Lanzhou China

**Keywords:** kidney neoplasms, perivascular epithelioid cell neoplasms, therapeutics, TOR serine–threonine kinases

## Abstract

Renal epithelioid angiomyolipoma (EAML) is a unique subtype of angiomyolipoma that contains a variety of cytoplasmic‐rich, eosinophilic cytoplasm epithelioid cells in addition to mature adipocytes, hyaline thick‐walled vessels, and smooth muscle‐like spindle cells. In recent years, increasing evidence has shown that EAML is a potentially malignant tumor. Due to the lack of typical clinical manifestations and imaging features, it is difficult to diagnose before surgery, and the diagnosis mainly depends on postoperative histopathological examination. With the advancement of pathological diagnostic techniques, more EAML cases has been discovered, but clinicians still lack a comprehensive understanding of EAML. This review comprehensively describes some pathological and clinical features of EAML, with special attention to the pathogenesis and treatment of malignant EAML in order to assist with clinical diagnosis and treatment.

## 1 INTRODUCTION

Perivascular epithelioid cell tumor (PEComa) is a general term for a class of mesenchymal tumors derived from different organs presenting with similar histomorphological and immunohistochemical expressions. The term was first proposed and named by Bonetti.^1^ According to the latest World Health Organization (WHO) classification criteria for soft tissue tumors (2020), PEComa includes angioleiomyomas, lymphangioleiomyomatosis, and non‐specific types of PEComa from soft tissues and viscera (PEComa‐not otherwise specified，PEComa‐NOS). Epithelioid angiomyolipoma (EAML) is a unique subtype of angiomyolipoma (AML) defined as a mesenchymal tumor of the kidney of unknown nature in the fifth edition of the WHO Classification of Tumors of the Urinary System and Male Reproductive Organs in 2016.^2^ Although the global incidence of EAML is low, studies have shown that this tumor has a different biological behavior from classic AML and has particular malignant potential, including local recurrence, invasion, and distant metastasis.

## 2 EPIDEMIOLOGICAL CHARACTERISTICS OF RENAL EPITHELIOID ANGIOMYOLIPOMA

Renal EAML is rarely encountered in clinical practice, with an incidence of approximately 1% for renal tumors and 4.6%–7.7% for renal AMLs.^3^ According to current literature reports, middle‐aged female patients generally have an insidious onset, presenting with insignificant clinical symptoms, and the renal tumors of these patients are often found during physical examination. Some patients, depending on the location and size of the tumors, may also have clinical symptoms such as fatigue, fever, low back pain, abdominal pain, hematuria, bleeding, dysuria, and renal dysfunction. Recently, Benincasa et al.^4^ reported a case of spontaneous renal rupture caused by PEComa, and although this is relatively rare in clinical practice, it should receive attention as the tumor is also known to cause paraneoplastic syndromes such as a high erythrocyte sedimentation rate, elevated platelet count, and hormonal and immune system abnormalities.^5^

## 3 ETIOLOGY AND PATHOGENESIS OF RENAL EPITHELIOID ANGIOMYOLIPOMA

**FIGURE 1:**
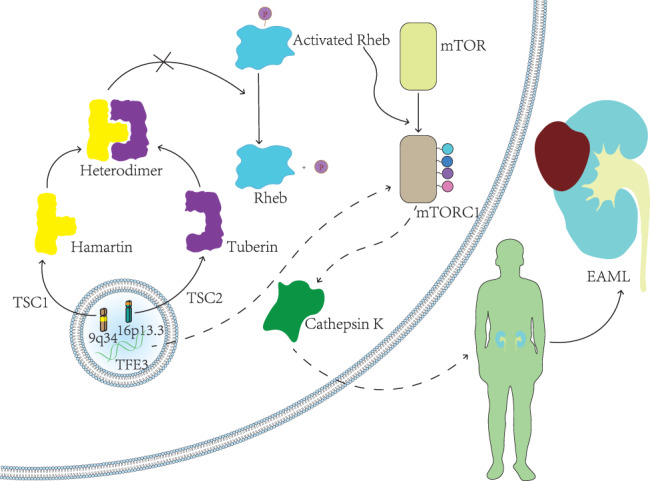
The potential pathogenesis of EAML. This figure shows two different pathogenesis of EAML. One possible mechanism is that *TSC*‐related gene mutations lead to impaired formation of the heterodimer complex encoded by it, followed by the abnormal activation of the mTOR pathway involved in tumorigenesis and the production of malignant biological behavior. Additionally, another scenario involves the translocation of the *TFE3* gene, which can also affect the activation of the mTOR pathway. EAML, epithelioid angiomyolipoma

Tuberous sclerosis complex (TSC) is an autosomal dominant disorder characterized by hamartomas of the brain, heart, skin, lungs, and kidneys.^6^ Studies have shown that a close relationship between renal AML and TSC exists. According to the latest follow‐up data of the TuberOus SClerosis registry to increase disease Awareness (TOSCA), more than half the patients with TSC have renal AML, and they have an increased risk for severe tumor rupture and bleeding, inevitably endangering their life. Thus, careful attention must be paid to the screening and treatment of AML in patients with TSC.^7^ Recently, genetic studies on renal EAML and TSC are receiving increasing attention. Abnormalities in the *TSC* gene have been detected in some patients with TSC primary disease and sporadic EAML, as shown by mutations in the *TSC1* gene on 9q34 or the *TSC2* gene on 16p13. *TSC1* and *TSC2* are tumor suppressor genes that encode for hamartin and tuberin, respectively. These genes interact to form heterodimers with GTPase activity. This dimer can dephosphorylate Ras homolog enriched in the brain (RHEB) and inactivate it, thereby inhibiting the formation of activated mTORC1, consequently impeding cell division and differentiation. Patients with primary TSC disease and sporadic EAML due to various causes have mutations in the *TSC1* and *TSC2* genes, resulting in the inability of hamartin and tuberin to form complexes, leading to an increase in RHEB‐GTP that binds to mTOR1 to form activated mTORC1. The activated mTORC1 then drives the mTOR/p70S6K metabolic pathway, which results in increased phosphorylated p70S6K, decreased protein kinase B (Akt) phosphorylation, reduced inhibition to cell differentiation, and, eventually, increased vascular smooth muscle differentiation and cell growth.^8^ Given the differences in pathogenesis, treatment, and prognosis between EAML and other renal tumors such as the most common benign renal tumor—AML and the most common renal malignant tumor—renal cell carcinoma (RCC), accurate diagnosis is key to achieving a favorable outcome. However, there are no specific tumor markers for EAML. The initial detection mainly depends on imaging examination, whereas the diagnosis depends on histopathology. With the advancement of molecular pathological diagnostic techniques, exploring the tumor‐related pathogenesis at the genetic level is vital for successful treatment and improved prognosis. Recently, Phillip et al. concluded through a cohort analysis that *TSC2* is the most common pathogenic factor of EAML, and *TP53*, *ATRX*, and *RB1* mutations are frequently noted in malignant EAML.^9^ However, currently, there are studies of small sample sizes where some of the listed genes were not detected. Therefore, further collection of samples and the detection of related genes are needed to further improve the understanding of the pathogenesis of EAML. Simultaneously, numerous reports have detected rearrangements of *TFE3* gene in malignant PEComa cases.^10^, ^11^, ^12^, ^13^ The *TFE3* gene product is a transcription factor of the MiTF/TFE family and helps regulate the gene expression of the transforming growth factor‐β signaling pathway. *TFE3* can participate in the regulation of cellular metabolism by stimulating lysosomal formation, changing the cellular response to oxidative stress, and increasing autophagic processes, leading to the activation of the mTOR signaling pathway, eventually promoting tumorigenesis.^14^ As seen in Figure 1, there are recent studies that have suggested that such tumors are a unique subtype of PEComa. However, in a multicenter study of 27 cases of PEComa with *TFE3* rearrangement, Chinese scholars recently found that the clinicopathological features, prognosis, and molecular genetic changes of PEComa with *TFE3* rearrangement were different from those of traditional PEComa. This suggested that such tumors should be classified as Xp11 translocation tumors with pigment differentiation.^15^ Notably, renal PEComa with Xp11 translocation can be readily confused with Xp11 translocation renal cancer due to their similarity in terms of nomenclature and gene mutation. However, these two tumors are intrinsically different as the former is a mesenchymal tumor that does not express epithelial markers and the latter is an epithelial tumor. Additionally, Renal PEComa with Xp11 translocation should be diagnosed and distinguished from Xp11 translocation renal cancer as it presents with a more aggressive biological behavior, and patients suffering from this have a poorer prognosis as they are prone to tumor recurrence, metastasis, and death.^16^

## 4 CHARACTERISTICS OF MALIGNANT RENAL EPITHELIOID ANGIOMYOLIPOMA

In 2000, Martignoni et al.^17^ proposed the concept of malignant EAML when they reported a primary lesion of renal EAML with postoperative pulmonary and abdominal metastasis. Scholars have successively recommended different diagnostic criteria for malignant PEComa based on existing case studies. Tsai et al.^18^ reported 23 cases of EAML, of which 5 (21%) showed aggressive behavior, including vascular invasion and distant metastasis. They classified cases of tumor thrombus and/or distant metastasis directly into the “aggressive group” and concluded that such patients require more cautious disease monitoring and early intervention during treatment. Brimo et al.^19^ analyzed 40 patients with EAML and found that one‐third of EAMLs showed invasiveness and concluded that the number of large epithelioid cells in the tissue, severity, degree of nuclear atypia, mitotic count, presence of atypical mitotic figures, necrosis, and presence of lymphovascular invasion could be used as a basis for differentiating between benign and malignant EAML. They proposed a predictive model in which EAML can be considered malignant if three or more of the following four conditions are met: (1) epithelial nuclear atypia ≥70%, (2) mitotic count ≥2 per 10 high‐power field, (3) presence of atypical mitotic figures, and (4) presence of necrosis. This predictive model was considered to predict 78% of malignant EAMLs. On the other hand, through follow‐up investigation of multicenter cases, Lei et al.^20^ concluded that the presence of three or more of the following characteristics predicted an increased likelihood of tumor malignancy: (1) tumor size >9 cm, (2) tumor thrombus formation in the vein, (3) epithelioid cells >70% or atypical cells >60%, and (4) necrosis. Table 1 summarizes the differential diagnosis between malignant EAML and benign EAML.

**TABLE 1:** The differential diagnosis between malignant EAML and benign EAML

	CT	Kidney biopsy
Malignant EAML	Tumor size usually large (>9 cm) Necrosis present Metastases present Extrarenal extension Formation of a cancer thrombus in a vein	Epithelial nuclear atypia ≥70% Mitotic count ≥2 per 10 high power fields Atypical mitoses Necrosis Epithelioid cells >70% atypical cells >60%
Benign EAML	Tumor size usually small	Epithelioid component represents>85% of the tumor

Abbreviation: EAML, epithelioid angiomyolipoma.

## 5 DIAGNOSIS OF RENAL EPITHELIOID ANGIOMYOLIPOMA

Typical AML is usually composed of a variable number of smooth muscle cells, malformed thick‐walled vessels, and mature adipose tissues. This can also be generally diagnosed by imaging studies. On the other hand, EAML is mainly composed of epithelioid cells or woven cells with little to no fat content. However, the reduced internal fat content makes it challenging to differentiate AML from RCC on imaging; thus, the final diagnosis relies on tumor immunohistochemical staining.

### 5.1 Imaging findings

**FIGURE 2:**
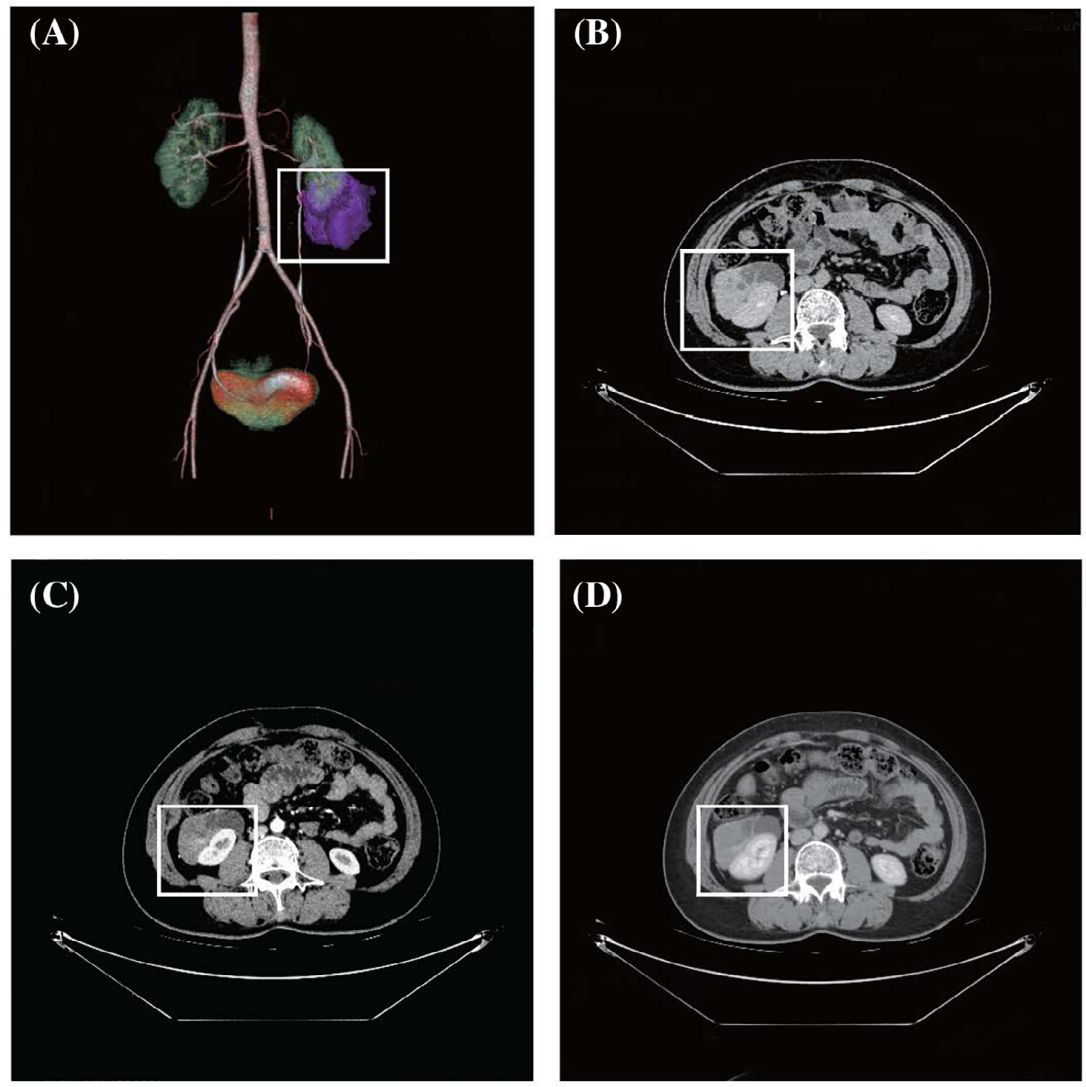
(A,B) A middle‐aged woman with EAML reported on a medical check‐up. Irregular shape, abutting the renal capsule, and indistinct boundary with the renal parenchyma were observed. (C,D) Contrast‐enhanced scan revealed heterogeneous enhancement with fast in and slow out. EAML, epithelioid angiomyolipoma

Ultrasonography is commonly used to screen for renal tumors due to its convenience and reproducibility. Typical AMLs contain fat components that usually appear as homogeneous hyperechoic lesions on ultrasonography, whereas EAMLs lack‐specific findings on plain ultrasonography. Contrast‐enhanced ultrasonography (CEUS) is a relatively new technique with unique advantages over traditional imaging modalities because of the absence of nephrotoxicity and ionizing radiation; furthermore, it has the ability to assess the enhancement pattern of renal lesions in real time.^21^ However, many studies have shown that CEUS cannot distinguish benign from malignant renal tumors.^22^, ^23^ Nevertheless, this method can still be used in daily clinical practice, such as in excluding renal pseudo‐lesions, inflammatory lesions and traumatic pseudo‐lesions and in differentiating between atypical renal cysts and renal cancer. Additionally, further computed tomography (CT) examinations are required in patients with abnormal ultrasonograms. The classic hallmark of AML on CT is the presence of fat density as it presents as a hypodense lesion with a CT value <−10 HU. In contrast, the EAML tumor volume is larger (usually >7 cm) relative to AML, and some patients may present with hemorrhage and necrosis. The CT findings of EAML are irregular, mixed‐density mass shadows (usually >45 HU) with heterogeneous enhancement. A solid or multilocular mass with insignificant enhancement on a contrast‐enhanced CT scan^24^ and “fast‐in and slow‐out” type on dynamic CT scan may also be observed. As seen in Figure 2, the appearance of this phenomenon in EAML may be related to its abnormal hypervascularity, higher cell density, reduced tumor matrix, the presence of intact tumor capsules, and the lack of tissue structure with reflux vessels.^25^ In contrast, the appearance of clear cell carcinoma is “fast‐in and fast‐out” type on a contrast‐enhanced CT scan. On magnetic resonance imaging (MRI), classic AML appears to be devoid of signal on fat suppression imaging.^26^ On the other hand, characteristic imaging findings on MRI of EAML mainly show hypointensity on T2‐weighted imaging, reticular enhancement, and “fast‐in and fast‐out” attributes.^27^ In addition, tumor size is another factor suggestive of EAML. While renal AMLs are small, EAMLs are usually large and are often associated with hemorrhage and necrosis. Patients with tumors >4 cm in maximum diameter have a 3.8‐fold higher chance of being diagnosed with EAML than the average patient.^28^ Table 2 summarizes the differential diagnosis between Classic AML and EAML. Due to the changes in the content of each component, EAML has no characteristic imaging findings, and some outcomes show overlapping findings with other renal tumors. Nevertheless, imaging examination has importance in formulating surgical plans and predicting a patient's prognosis as this may aid in terms of finding the location of the tumor and determining the structures proximal to it.

**TABLE 2:** The differential diagnosis between classic AML and EAML

	US	CT	MRI
Classic AML	Fatty components and usually appear as homogeneous hyperechoic lesions	Low‐density lesions with CT value <−10 HU	Hyperintense on both T1‐ and T2‐weighted images and appear hypointense on fat suppressed T1 images
Epithelioid AML	Lack of specific manifestations	CT shows irregular mixed density mass shadows (usually >45 HU) with heterogeneous enhancement, “fast‐in and fast‐out” type on a contrast‐enhanced CT scan	T2‐hypointense，reticular enhancement, and “fast‐in and fast‐out” attributes

Abbreviations: AML, angiomyolipoma; EAML, epithelioid angiomyolipoma; US, ultrasound.

### 5.2 Histopathological examination

**FIGURE 3:**
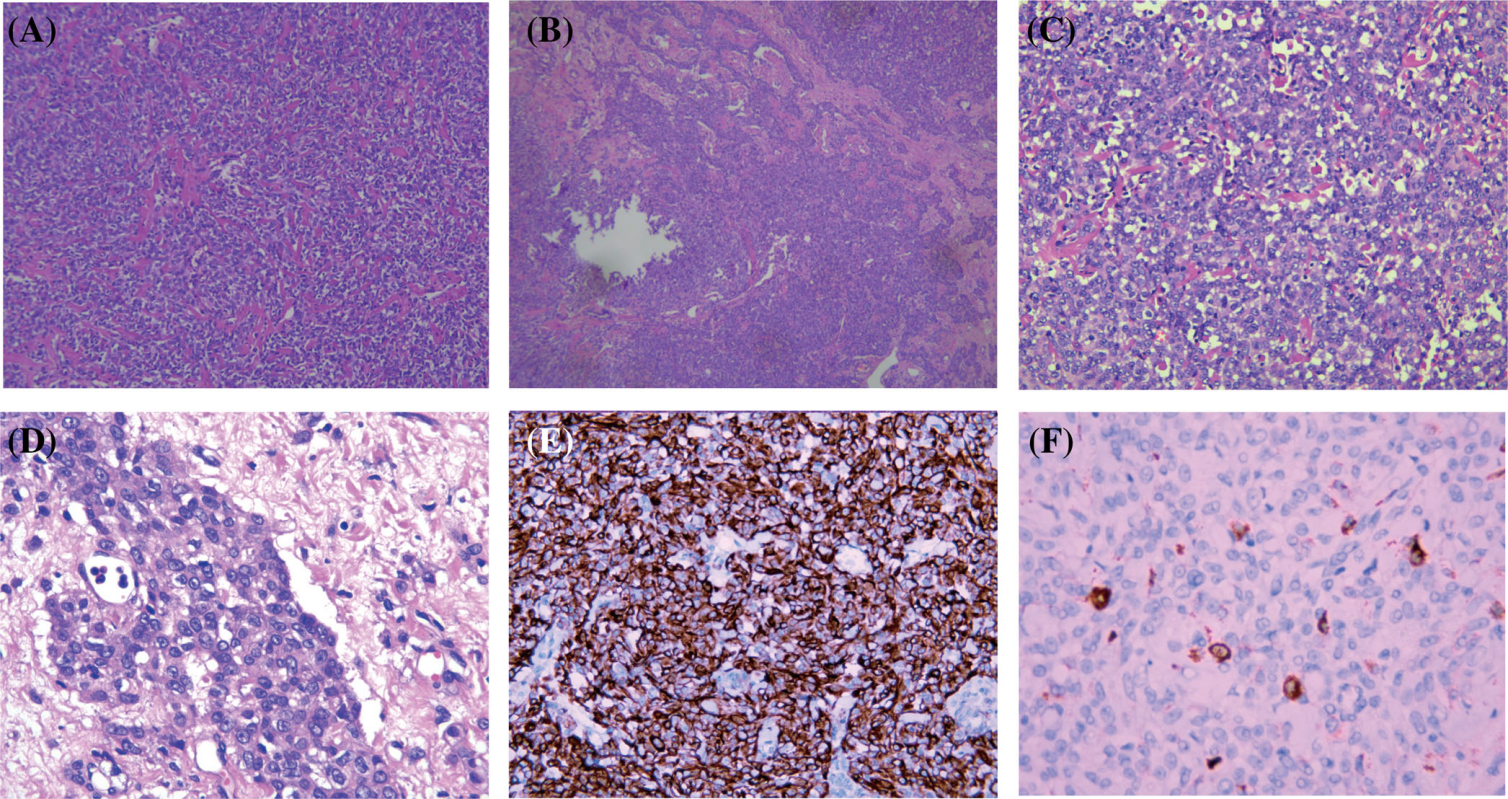
(A–C) A hematoxylin–eosin staining specimen of EAML shows both epithelioid components including epithelioid cells with an intercellular bridge and smooth muscle component with little fat. (D) EAML with positive HMB‐45. EAML, epithelioid angiomyolipoma

In the absence of essential imaging evidence, a pathological examination is required to further confirm the diagnosis in cases that are difficult to confirm radiologically. Histopathological examination, including needle biopsy and immunohistochemical examination of surgical specimens, can be further used to confirm the diagnosis. As seen in Figure 3, there are a variety of cytoplasmic‐rich, clear‐to‐eosinophilic cytoplasm epithelioid cells in addition to mature adipocytes, hyaline thick‐walled vessels, and smooth muscle‐like spindle cells. However, there is no uniform standard in the academic community in terms of establishing the number of epithelioid cells needed to conclusively diagnose EAML. The current inclusion criteria vary from 10%^29^ to 95%,^30^ while the WHO recommends epithelioid cells greater than 80% as the diagnostic criteria for EAML. A more precise diagnostic criteria is beneficial to identify the characteristics of malignant EAML as this can serve as a guide to clinical treatment. Fine needle aspiration biopsy (FNA) is a minimally invasive and cost‐effective test that is usually used as the initial diagnostic modality to assess mass lesions. However, due to sampling limitations, especially when epithelioid components account for the majority, it is not possible to make a definitive diagnosis.^31^ Azawi et al.^32^ assessed the accuracy of core needle biopsy (CNB) in 129 solid renal masses ≤4 cm in size and found that CNB had good accuracy and specificity when the mass was ≤4 cm; however, EAML is usually large, which may affect the accuracy of a CNB‐based diagnosis. Surgical specimen examination is required to guide treatment for pathologies that cannot be identified by needle aspiration cytology. EAML is nodular or irregularly oval in gross view, soft in texture, partially cystic in appearance, and has a grayish‐yellow or grayish‐brown cut surface and pseudo‐capsule. On the other hand, epithelioid tumor cells are arranged radially around the hyalinized vascular wall, have abundant cytoplasm, have clear or eosinophilic staining, vacuolated nuclei, and are sometimes accompanied by significant nucleoli and necrotic and pathological mitotic figures. Immunohistochemical staining shows a positive reaction to melanoma‐associated markers such as SOX10, HMB‐45, HMB‐50, Melan A, MITF, and NKI‐C3 and myogenic markers like SMA, but not to epithelial markers such as cytokeratin.^33^, ^34^ Compared to EAML, RCC is positive for epithelial markers but negative for melanocytic markers.^25^ Recently, it has been documented that the “conventional pan‐melanoma cocktail” can be used to diagnose EAML because the melanocyte markers HMB‐45, Melan‐A, SOX10, and tyrosinase can be expressed in some EAML, and because S100 is usually not expressed in EAML, it can be used to differentiate from melanoma, but this method requires further testing.^35^, ^36^, ^37^, ^38^ In addition to the classic immunohistochemical markers mentioned above, specific immunohistochemical markers of PEComa have been explored in the recent years, and it has been found that cathepsin K and at least one muscle marker are expressed in all PEComas.^39^ This is consistent with the results of Caliò et al.'s study that suggested that cathepsin K may be related to TFE3 rearrangement and mTOR pathway activation and may be used as a target for drug therapy in the future.^40^ Caliò et al. also proposed that parvalbumin is expressed in many cases and can even be used as an immune marker for EAML lesions.^41^ Moreover, PNL2 has been shown to be a biomarker of sensitivity and specificity in melanoma; recently, some scholars have found that compared with HMB‐45, PNL2 has a higher sensitivity for differentiating PEComa from other renal tumors (85% vs. 81%) and has specificity in malignant PEComa (89% vs. 81%).^42^

## 6 TREATMENT OF RENAL EPITHELIOID ANGIOMYOLIPOMA

Due to the absence of evidence from prospective trials as well as the absence of established treatment guidelines, the treatment of malignant PEComas can become very complicated. Currently, reported treatment options include surgical treatment, chemotherapy, targeted therapy, immunotherapy, and endocrine therapy.^31^ Surgical resection is the mainstay of treatment for renal EAML,^43^ and the surgical approach can be determined according to the RENAL score. For tumors ≥4 cm in size, radical nephrectomy is considered the ideal treatment. In contrast, for tumors <4 cm, partial nephrectomy is usually performed. Because it is difficult to differentiate EAML from renal cancer using preoperative imaging, an intraoperative frozen rapid pathological examination is generally used. If malignancy or marked cellular atypia cannot be ruled out, partial nephrectomy or radical nephrectomy is feasible. However, some patients still experience local recurrence or distant metastasis after surgery. In addition, interventional therapy, including selective arterial embolization (SAE), ablation and SAE combined with ablation, has become an important treatment option, especially for patients with acute rupture with hemorrhage and those intolerant to surgery. Currently, it is believed that interventional therapy should be considered when the maximum diameter of renal AML is >4 cm or relevant clinical symptoms appear.^44^ However, postoperative renal impairment, tumor recurrence, and retreatment after embolization remain urgent problems to be solved in clinical practice. In this challenging context, standard chemotherapy regimens for soft tissue sarcomas have only been shown to be effective in a small subset of patients, and long‐term results remain unsatisfactory.^45^ Mutations in the *TSC1*/*TSC2* gene and translocation of the *TFE3* gene leading to the hyperactivation of the mTOR complex are characteristic of this tumor. Recently, some scholars have also speculated from the molecular genetics of PEComa that inhibition of mTOR activity using mTOR inhibitors such as rapamycin and everolimus can control tumors.^46^ Many clinical systematic reviews have demonstrated that these drugs cause the primary tumor and metastases to shrink in size and that disease and complications are effectively controlled. However, due to the presence of changes in the mTOR pathway, patients must continue taking the drugs, and there may be cases of resistance to mTOR inhibitor therapy. Moreover, Peces et al. found that low‐dose rapamycin reduced the tumor volume and delayed the loss of renal function in patients with tuberous sclerosis and renal PEComa disease.^47^ The mucosal toxicity of sirolimus can be reduced using personalized treatment regimens based on the detection of sirolimus blood level drug concentration and assessment of clinical tolerance.^48^ Although changes in the mTOR pathway respond well to mTOR inhibitors, some patients still experience drug resistance or disease progression. Prospective studies are therefore urgently needed to determine second‐line treatment options for such cases. Due to the close relationship between the occurrence of PEComa and blood vessels, a few case studies have found that angiogenesis inhibitors (such as bevacizumab, pizopanib, and sorafenib) alone or in combination help eliminate the primary tumor and distant metastasis of tumors for advanced PEComa.^45^, ^49^ For TFE3‐rearranged malignant PEComas, mTOR inhibitors have a limited therapeutic effect, and targeting VEGF/VEGFR signaling may be a new and effective therapeutic option for TFE3‐associated malignant PEComas.^50^ Another retrospective series reported reversal of resistance to mTOR inhibitors after adding exemestane to the treatment regimen of advanced PEComas, suggesting that combining mTOR inhibitors and exemestane will be effective against tumor refractory to mTOR inhibitor therapy alone. The combination therapy with mTOR inhibitors and exemestane is only effective against TSC1/2‐mutated PEComas. Further studies are required to explore whether it is effective against TFE3‐associated PEComas.^51^ PD‐1^+^ T cell infiltration and high expression of PD‐L1 have been detected in PEComa specimens using immunohistochemistry. Administering PD‐1 antibody or PD‐1 antibody combined with the cytotoxic T‐lymphocyte‐associated antibody antigen‐4 in *TSC* gene‐deficient mice increased T cell infiltration in tumor tissues and effectively inhibited tumor growth.^52^ Maisel et al.^53^ similarly detected upregulation of PD‐L1 in pulmonary lymphangioleiomyomatosis. In a mouse model, anti‐PD‐1 antibody treatment increased T lymphocyte infiltration and significantly prolonged survival. Lattanzi et al.^54^ first reported a case of malignant EAML with *TSC* gene mutation and resistance to mTOR inhibitor therapy. The patient's condition was effectively controlled after switching to PD‐1 antibody (nicotine) treatment, suggesting that the PD‐1 antibody is expected to be a breakthrough against malignant EAML. With the rapid emergence of personalized treatment for tumors and immunotherapy, more data from clinical cases and evidence‐based trials must be accumulated to prove its effectiveness for malignant EAML.

## 7 CONCLUSIONS

EAML is a tumor of intercellular origin that expresses both myogenic and melanin markers, and there is increasing evidence that it has some malignant potential. Unlike AML, EAML contains a certain number of epithelioid cells that may be associated with its malignant behavior. However, the number of epithelioid cells in the diagnostic criteria regarding EAML has not been established. Additionally, in imaging and histopathological examinations, EAML can be easily confused with other tumors, especially RCC, which is the most common malignant tumor of the kidney. Surgical resection is the primary treatment, and considering the malignant potential of renal EAML, long‐term follow‐up is required after the surgery. Aberrant activation of the mTOR pathway is its key pathogenesis and may contribute to the development of its malignant behavior. Targeted mTOR therapy can be used as first‐line treatment in patients who cannot be treated by surgery. At present, most of the research on EAML is based on case reports or analysis, and there is still a lack of comprehensive understanding of its clinical and pathological features. With the advancement of molecular pathological diagnostic techniques and the arrival of the era of cancer immunotherapy, we can clearly diagnose, treat, and manage EAML, thereby impacting the prognosis of the patients.

## CONFLICT OF INTEREST

The authors declare no conflict of interest.
